# The Acceptability and Usefulness of Cognitive Stimulation Therapy for Older Adults with Dementia: A Narrative Review

**DOI:** 10.1155/2016/5131570

**Published:** 2016-07-11

**Authors:** Hui Moon Toh, Shazli Ezzat Ghazali, Ponnusamy Subramaniam

**Affiliations:** Health Psychology Programme, School of Healthcare Sciences, Universiti Kebangsaan Malaysia, Raja Muda Abdul Aziz, 50300 Kuala Lumpur, Malaysia

## Abstract

Cognitive stimulation therapy (CST) is an evidence-based therapy for individuals with mild-to-moderate dementia. Past reviews have only synthesized outcomes obtained through quantitative study which does not fully represent the understanding on the acceptability and usefulness of CST. Therefore, the present review aims to integrate outcomes obtained from both quantitative and qualitative studies to provide a deeper understanding on the acceptability and usefulness of CST for older adults with dementia. Findings of literature were retrieved from searches of computerized databases in relation to CST for people with dementia. Literatures were selected according to selection criteria outlined. Results obtained in previous studies pertaining to the effects of CST were discussed in relation to variables such as cognitive function, quality of life, and family caregivers' wellbeing. The review also explores the use of CST in different cultural context, the perception on its effectiveness, and individualized CST (iCST). There is considerable evidence obtained through quantitative and qualitative studies on the usefulness and acceptability of CST for older adults with dementia. Recommendations for future research are provided to strengthen the evidence of CST's effectiveness.

## 1. Introduction

Dementia is a neurocognitive disease characterized by progressive, global deterioration in intellectual abilities including memory, learning, orientation, language, comprehension, and judgement. Currently in the DSM-5, dementia is referred to as Major Neurocognitive Disorder [1]. According to Alzheimer's Disease International [2] in a systematic review of the global prevalence of people with dementia above 60 years old, identifying 147 studies in 21 Global Burden of Disease (GBD) world revealed that the regions which have the highest prevalence were observed in Latin America (8.50%) and the lowest in East Asia (4.98%). The review also yielded an estimation of the prevalence of people with dementia across 21 GBD regions. According to the estimation, 35.6 million people worldwide will be living with dementia in 2010 and the number will increase almost twofold every 20 years, to 65.7 million in 2030 and 115.4 million in 2050. Hence, as of 2013, there will be estimated 44.4 million people with dementia worldwide and most of them will be living in developing countries like China, India, south Asian, and western Pacific regions.

The numbers of people with dementia grow as the elderly population increases. Therefore, effective interventions are highly sought after to alleviate the negative impact of dementia has on the person. Both pharmacology and nonpharmacology intervention are common for people with dementia. The term nonpharmacology treatments for people with dementia usually refer to psychosocial interventions. One of the popular psychosocial approaches is cognitive-based intervention which includes cognitive training, cognitive rehabilitation, and cognitive stimulation [3]. Present review will focus on interventions that are based on cognitive stimulation. Clare and Woods [4] defined cognitive stimulation as “*an engagement in a range of activities and discussions (usually in a group) aimed at general enhancement of cognitive and social functioning.*”

Cognitive stimulation therapy (CST) is a brief, evidence-based intervention for people with mild to moderate dementia [5]. The intervention is usually conducted in group involving 14 sessions of themed activities such as current affairs, word associations, and money. The intervention does not aim to test factual answers but to encourage participants to give their opinions and thus to actively stimulate and engage them in an optimal learning environment, usually with the social benefits of a group. The intervention can be conducted by trained individuals to work with people with dementia with the aid of CST manual or CST training. The intervention can be conducted in various settings including residential homes, care homes, day care centres, and memory clinics. Longer-term or maintenance CST (mCST) is also available following the brief CST. In addition to the brief CST which is usually conducted in group by trained CST facilitators, individual cognitive stimulation therapy (iCST) has been developed to be delivered by family caregivers to the people with dementia individually at home.

While systematic literature review on CST has been published [6], the focus of the review remains mainly on quantitative studies. Thus, the Cochrane review has only included studies using randomized controlled trials (RCTs). Therefore, little emphasis was given to the outcomes obtained through qualitative studies which might be crucial in understanding the effectiveness of CST and its perceived effectiveness. Hence, the present review aims to evaluate the existing literature from both qualitative and quantitative studies on the acceptability and usefulness of cognitive stimulation therapy (CST) for older adults with dementia.

## 2. Methods

### 2.1. Selection Strategy

A search was conducted in August 2015 across various electronic databases including PubMed, Springer Link, Science Direct, and Wiley Online Library. The search terms used were “cognitive stimulation therapy” AND “dementia” in Title, Abstract, and Keywords.

### 2.2. Selection Criteria

Only studies that focused primarily on cognitive stimulation therapy on people with dementia were included for review. Studies that combined CST with other modalities such as medication and studies that focus on maintenance cognitive stimulation therapy (mCST) were excluded. Studies were not excluded based on demographics of research participants, methodology, assessment tools, outcome measures, or results. The scope was limited to studies in English, published in peer-reviewed journals between 1990 and August 2015 and available in full original text.

### 2.3. Data Extraction/Collection

The PubMed searches yielded 31 references for consideration, and of these, 11 were included, Springer Link yielded 6 references, 3 of which were included, Science Direct yielded 4 references, 1 of which was included, and Wiley Online Library yielded 5 references, 2 of which were included. A total of 28 references were excluded from this review, 9 of which were focusing on mCST, 8 of which were a review paper, 4 of which were commentaries and letters, 3 of which were a study that combined CST with other therapies (i.e., drug therapy and carer training program), 2 of which were in press, 2 of which were irrelevant in content (i.e., computer models in evaluating efficacy of CST, and 1 of which was a step-by-step guideline). There are 5 duplications in the studies included across the databases. Thus, upon removing the duplicated studies, present paper will only review on 12 studies.

## 3. Results

The results of the literature search yielded 12 relevant studies. All articles were reviewed and summarized using a standard data extraction form inclusive of study sample, assessment tools, research methodology, research outcomes, and clinical implications if available (Table 1).

### 3.1. CST and Cognition

Spector et al. [7] used a randomized control trial (RCT) to determine the effects of CST on different areas of cognitive functions for people with dementia. In order to achieve this objective, ADAS-Cog was examined in detail. ADAS-Cog includes items such as word recall, naming, commands, constructional praxis, ideational praxis, orientation, word recognition, spoken language, comprehension, word-finding, and remembering instructions. These items can be combined to form three subscales, namely, memory and new learning, language, and praxis. Results showed that the items “commands” and “spoken language” showed significant difference favoring the intervention group and among the three subscales, intervention group improved significantly over the control group in language subscale.

Besides language, study by Hall et al. [8] also found that people with dementia who underwent CST had remarkably improved in memory, comprehension of syntax, and orientation. These participants performed significantly better in neuropsychological tests such as delayed verbal recall, visual memory, and auditory comprehension. It was hypothesized that the improvement in comprehension of syntax is attributable to the language-based nature of CST.

Additionally, Aguirre et al. [9] highlighted the influence of demographic variables on the effects of CST on cognition of people with dementia. The research outcomes indicated that age is a significant predictor of the study outcome. There are greater effect on the post-MMSE score and ADAS-Cog score among older participants in the study (older than 80 years) as compared to the participants from younger age group which showed little difference in pre- and post-MMSE and ADAS-Cog score. Besides, the outcome also indicated gender as a significant variable in the study. Using ADAS-Cog scores, female participants showed greater cognitive improvement as compared to male participants.

### 3.2. CST and Quality of Life (QoL)

A single-blind multicentre RCT study on 201 participants with dementia by Spector et al. [10] showed improvement in intervention group in quality of life. QoL in the study was measured using Quality of Life-Alzheimer's Disease (QoL-AD). QoL in the study showed significant improvement in intervention group which received CST but deteriorated in control group which perform usual activities.

Although the significant improvement in QoL through participation in CST was noted in preceding studies, the underlying mechanisms in the improvement of QoL after CST intervention were not properly examined. Hence, Woods et al. [11] have conducted a RCT study to examine whether the effect of CST on QoL was mediated by the changes in cognition and whether there are any specific domains of quality of life which change in response to the intervention. The results showed that increase in QoL correlated significantly with improvement in cognition as measured by MMSE and ADAS-Cog, with the reduction of symptoms of depression as measured by Cornell Scale for Depression in Dementia (CSDD) as well as with the improvement of communication abilities as measured by Holden Communication Scale (HCS). Since correlation was not identified between QoL and cognitive function at baseline level, multiple regression analysis was utilised to evaluate whether change in cognition (MMSE) in postintervention accounts for change in QoL. The outcomes of the study concluded that although QoL is independent of cognition, intervention to improve cognition also can improve QoL.

Demographic variables have also played its role in the effects of CST on QoL. Woods et al. [11] have identified gender as a significant predictor of changes in QoL. The outcomes suggested that the significant improvement in QoL was associated with female as compared to male. Aguirre et al. [9] also identified living situation as an important variable in studying QoL. The study outlined an increased in mean score of Dementia Quality of Life (DEMQOL) Proxy in both community-based and care home-based participants in the study. However, the change in score in the care home group was noted larger than the change in community-based group.

### 3.3. Perception on CST

As mentioned earlier in this paper, previous studies clearly illustrated the effectiveness of CST in improving cognition and QoL in person with dementia. Spector et al. [12] further investigated whether the improvements found in the studies were noted by the participants, their caregivers, and group facilitators involved using qualitative study. Information gathered from qualitative interviews and focus group was analysed using Framework Analysis.

Through analysis, two main themes emerged across all three groups of participants which are “positive experience of being in the group” and “changes experienced in everyday life.” Under the theme “positive experience of being in the group”, four subthemes were identified as follows: (i) positive feelings were experienced during the CST group as it made them feel more positive, relaxed, and confident and that they wanted to continue with the group; (ii) listening to others and feeling able to talk contrasted the feelings of loneliness and a passive state of mind that emerges when they were alone at home; (iii) sharing a diagnosis made them realise that they are not alone and this made them felt supported; and (iv) the supportive and nonthreatening environment in CST group create opportunity to get support and also to provide support to others. Meanwhile, three subthemes were identified under the theme “changes experienced in everyday life” as (i) finding talking easier in a group environment during CST and some showed greater willingness to engage in conversation, (ii) improvement in memory, and (iii) improvement in concentration and alertness.

### 3.4. Cost Effectiveness of CST

In order to provide CST service to the public, the therapy not only must be effective but also needs to be cost effective to be carried out in large scale. Knapp et al. [13] have investigated the cost effectiveness of CST intervention for people with dementia as part of RCTs. Results obtained from the study showed minimal differences in costs between the intervention group that received CST and the control group that received treatment as usual (TAU). Pertaining to the outstanding outcomes of CST in cognition improvement and QoL improvement, CST appeared to be more cost-effective than TAU. Besides, study done by Spector et al. [10] found that the CST yielded improvements that are comparable to improvement in person with dementia undertaking medication, specifically acetylcholinesterase inhibitor (AChEI) which is a common medication for treating dementia. This finding indicated that CST might be also more cost-effective than pharmacological treatment.

### 3.5. CST in Different Culture Context

Previous studies have unanimously pointed out that CST has been shown to be significantly beneficial in improving the cognitive function and QOL in people with dementia [9–11, 14]. However, these indicative outcomes were derived entirely from RCT studies conducted in United Kingdom. Considering cultural differences, Yamanaka et al. [15] replicated the single-blind, controlled clinical trial done by Spector et al. [10] through a Japanese version of CST, named CST-J. Cognitive stimulation therapy-Japanese version (CST-J) translated CST into Japanese language and also modified its contents to suit Japanese culture. For example, in the word games session, “Shiritori,” which is a traditional word-chain game, was used in replacement of crossword puzzles which were not very familiar in Japanese culture.

The results of the study were found to be comparable to the outcomes of previous CST studies in the United Kingdom. Similar to the intervention group who participated in the CST in United Kingdom, the intervention group in Japan that participated in CST-J also showed significant improvements in cognitive function and QOL as compared to the control group. Yamanaka et al. [15] proposed that although the contents within CST were amended to suit the Japanese culture, CST remains effective for people with dementia. In conclusion, CST is able to show promising benefits for people with dementia outside of the British context in which the therapy was developed. Yet, it is crucial that sufficient modification is performed to amend the contents into culture-appropriate contents before adopting CST in a different cultural setting.

Aside from UK and Japan, Dotchin et al. [16] have recently carried out a pilot study using CST in two sites in Sub-Saharan African (SSA) due to the pressing needs for intervention for elderly Africans who have dementia. The study obtained positive feedbacks from both person with dementia and their carers. Since it was a pilot study, the sample was not large enough to indicate significant changes in cognitive functioning. However, provisional results indicated small improvements in quality of life among the participants. The study has proven feasibility of CST in Africa continent which further suggests the adaptability of CST in different cultures.

### 3.6. CST and Individualized CST (iCST)

Due to its effectiveness, group CST is practiced widely in the United Kingdom. However, Orrell et al. [14] noted that there are still numerous people with dementia who may be unable or unwilling to participate in group CST. Hence, this led to the ideation of iCST which sessions are performed by the family caregiver at home for people with dementia. Orrell et al. [14] proposed several reasons older adults with dementia might have for not participating in group CST which may include the following: they do not want to go out, their restricted mobility or health issues prevent them from getting out; they choose not to participate in group-based activities, or groups are not available in their local area.

Aside from participation issues, a number of previous studies also demonstrate efficacy of the use of cognitive stimulation intervention in home environment, which further support the ideation of iCST. Home-based cognitive stimulation intervention involving the family caregiver showed significant improvements in cognition in person with dementia as well as improvements in caregivers' wellbeing [17–20]. Moniz-Cook et al. [17] also found reduction in care home admissions at 18-month follow-up after the intervention.

Prior to implementing or promoting iCST to the public, Orrell et al. [14] designed a multicentre, single-blind, randomized, two-intervention arm (iCST over 25 weeks versus intervention as usual, or TAU), controlled clinical trial to prove the effectiveness of iCST with 306 participants recruited from the community settings such as memory clinics, day centres, and Alzheimer's Society. Based on previous research findings, the researchers hypothesize that people with dementia receiving iCST will show improvements in cognition and quality of life. The clinical trial is still ongoing at present (August 2015).

In order to enhance the feasibility and suitability of iCST, a qualitative study by Yates et al. [21] was conducted. The study has suggested that the iCST was well received by people with dementia as well as their carer. Both people with dementia and carers reported positive perception of iCST and the importance of mental stimulation was well recognized. In terms of feasibility of the program, the study found that the feasibility of the program was much depending on whether the carer views practical issues such as finding time to deliver the program as insoluble barriers or issues that could be resolved. These preconceptions of the carer could be barriers towards effective implementation of the iCST. Nevertheless, this study has contributed useful information for program developer to further enhance and refine the program.

### 3.7. CST and Caregivers for Older Adults with Dementia

Schulz and Sherwood [22] suggested that the dominant conceptual model to date for caregiving is the stress-coping model which assumes that the onset and progression of chronic illness and physical disability are stressful for both the person with dementia and the family caregiver. Hence, it is expected that the positive outcomes of CST which improve the cognition and QoL in people with dementia will lead to an improvement in family caregivers' mood and wellbeing. In relation to this expectation, Aguirre et al. [23] conducted a study aiming to examine the impact on the family caregivers of the CST intervention for people with dementia. The study gathered information from 85 family caregivers of people with dementia who live in the community and currently participating in maintenance CST (mCST) intervention. The outcomes yielded did not adhere to the stress-coping theoretical framework. The outcomes illustrated no significant evidence of indirect effects of CST on caregivers' general health status and QoL.

Aguirre et al. [23] further discussed the plausible explanation for the challenged results. One of the possible reasons might be that the perceived health of the family caregivers is related not only to cognitive impairment of the person with dementia, but also to the behavioural and psychological symptoms in dementia as illustrated in the study by Donaldson et al. [24]. Besides, another possible reason might be that the influence of therapy on caregivers' wellbeing might depend on the nature of the relationship between the caregiver and the care recipient in the therapy. Hence, iCST which involves the caregiver in delivering the therapy might show greater effects of CST on caregivers' wellbeing. Previous home-based cognitive stimulation intervention which required family caregiver to deliver the intervention had elicited evidence of improvement in the caregivers' wellbeing [17, 18].

## 4. Discussion

This paper reviewed the acceptability and usefulness of cognitive stimulation therapy (CST) for individuals with dementia. Considering the amount of studies included in this paper, there is clearly a need for additional research in the use of CST in different cultural setting and in the use of iCST. Out of the 12 studies reviewed, only two studies were conducted outside of United Kingdom [15, 16] and only one study focused on iCST [14]. Although there is growing evidence of the effectiveness of CST, some evidence was drawn from short-term studies [8, 9, 14–16] which employed pretest and posttest design without follow-up assessment. The results of these studies did not provide insight into the long term effects of CST. Hence, longitudinal researches need to be implemented to warrant the lasting effect of the intervention.

Previous studies have provided sufficient evidence for the effectiveness of CST in improving cognition and QoL in older adults with dementia. The recent Cochrane review on CST [6] combined the data from 15 randomized controlled trials (RCTs) (*n* = 178) on people with dementia, showing significant effects of intervention over control conditions in cognitions as measured by the Mini-Mental State Examination (MMSE) and the Alzheimer's Disease Assessment Scale-Cognition (ADAS-Cog). The yielded outcomes are similar to the findings yielded in previous systematic reviews which showed indication of improved cognition using CST [25–27].

In relation to cognitive improvement, study in detail showed that CST improved language dimension of cognition in people with dementia and this result is being explained as an effect of the nature of CST which emphasizes implicit learning over explicit learning which focuses on rehearsal of information. Implicit learning in the CST context implied that, subsequent to the stimulation using materials, participants are encouraged to generate new views and opinions rather than factual answers, and establishment of new semantic links is constantly being encouraged as well. Emphasis on verbal communication accompanied by the opportunity to engage in meaningful conversations can reasonably improve language dimension in cognitive area. As the language dimension improves, communication between the person with dementia and people around him or her will improve subsequently. Improvement in communication of thoughts and feelings might serve as an indicator of changes in QoL following improvement in cognitive function. Later, in support to this assumption, study showed evidence that changes in cognition did not directly improve QoL but serve only as a mediator to improve QoL. This finding clearly indicates that the CST intervention which aims at improving cognition improves QoL in people with dementia as well [11].

Aside from research conducted in the Western context, in this case which is in United Kingdom, study of CST performed in Asian context yielded similar result. This is especially important to highlight the adaptability of CST into different culture while remaining its effectiveness in improving cognition and QoL. However, its adaptability in a different culture must be considered carefully. In the study by Yamanaka et al. [15], although the overall improvement in cognition and QoL is parallel to the original study [10], the self-rated QoL-AD was found to be insignificant in showing effective result which was different from the significant result obtained in the Spector et al. [10] study. The researchers attributed the differences in outcomes between the studies to the prominent characters of Japanese. Previous studies have shown that the Japanese generally practices self-critical and other enhancing biases in the interdependent construction of self, which could be related to Japanese low wellbeing [28, 29]. Hence, it is difficult for the participants of CST-J to show improvement in QoL in such a short-term intervention. In conclusion, the implication of a measure might be interpreted differently in a different culture and failure to do so can easily serve as culture biases.

Theoretically, wellbeing of family caregivers for people with dementia will increase alongside the improvement in cognition and QoL [22]. However, study by Aguirre et al. [23] failed to provide evidence for this assumption. Yet, it is not overall conclusive to state that this assumption is entirely incorrect. This is due to the fact that findings in a qualitative study by Spector et al. [30] on perception of people with dementia and their caregivers on CST intervention were generally positive feedbacks. Most caregivers noticed the positive changes in the person with dementia after CST and looking at the vignette recorded in the study, the caregivers are mostly delightful to share the positive changes they had witnessed in people they cared for. Hence, it is questionable to claim that CST brings no changes in the caregivers' wellbeing by only using quantitative measure which sometimes might not be sensitive enough to detect changes. In addition, an individual's wellbeing can be illustrated through a variety of expressions such as the changes in the level of anxiety, level of depression, and level of burnout. Therefore, it is much needed to measure a wide range of family caregivers' outcomes in order to get a clear picture on the effects of CST had on caregivers' overall wellbeing. The psychological tools used to measure these outcomes must also be sensitive to detect minor changes after the intervention. It is also probable that the intervention using CST involves fewer caregivers and hence the results may not be carried over to the caregivers. Thus, it is likely that the use of iCST can illustrate a clearer picture of the impacts of CST has on the caregivers' wellbeing as iCST requires forming of therapeutic relationship between the caregivers and the people receiving care.

Knapp et al. [13] have shown evidence of cost effectiveness of CST intervention which increases the perceived effectiveness of CST. The cost-effectiveness analysis was evaluated based on a range of values for decision-makers' willingness to pay for an additional point improvement on the MMSE and QoL. Although the analysis revealed that the family caregivers who make financial decision would be likely to view CST as a comparatively cost-effective option, it was analysed using a short-term CST intervention which might not be fair and accurate to be used to gauge its cost effectiveness in the long run. Therefore, it will be more conclusive that CST is a cost-effective psychological therapy if the cost effectiveness is measured in a longer term such as in mCST.

### 4.1. Limitations

The present review only includes studies in English which were published in peer-reviewed journals between 1990 and 2015 that have keywords of “Cognitive Stimulation Therapy” AND “dementia” and available in full original text. Therefore, the selection of studies to be included in the paper might has limited the access to other important and relevant studies that do not fit in the selection criteria. There is possibility of bias tendency in the review paper as only the available studies were included and reviewed.

### 4.2. Implications for Future Research

Overall, most studies reviewed in this paper have shown positive results of CST. However, these studies did not provide information on the distinction in effects of CST on people with different severity level of dementia. Hence, further research is needed to investigate the differences in effects of CST on people with different severity level of dementia. There is also a need for more CST research to be conducted in different cultural setting aside from United Kingdom in order to provide more evidence of its adaptability and usefulness in different cultures. Last but not least, studies should also compare the effectiveness and cost effectiveness between CST and iCST in attempt to provide the most suitable intervention for each individual with dementia accordingly.

## 5. Conclusion

Cognitive stimulation therapy (CST) can be an effective intervention for older adults with dementia to improve their cognitive functioning as well as QoL. In addition to the usefulness of CST and iCST, present review gauges the acceptability of CST in different cultural context. Given the inclusion of qualitative studies in this review, the usefulness and acceptability of CST also take into consideration the perspectives of the person with dementia, their family caregivers, and facilitators in care homes. Taking into account both quantitative and qualitative studies, future systematic review can provide an in-depth understanding on the usefulness and acceptability of CST in older adults with dementia.

## Figures and Tables

**Table 1:** Overview of the studies reviewed in this paper.

Study	Purpose	Design and sample	Outcome measures	Results	Conclusion(s)
Spector et al. [10], “Efficacy of an Evidence-Based Cognitive Stimulation Therapy Intervention for People with Dementia: Randomised Controlled Trial”	To test the hypothesis that CST for older people with dementia would benefit cognition and quality of life	Single-blind, multicentre RCT with baseline, postintervention, and follow-up assessments *n* = 201 Intervention = 115 Controls = 86	Mini-Mental State Examination (MMSE); Quality of Life-Alzheimer's Disease (QoL-AD); Alzheimer's Disease Assessment Scale-Cognition (ADAS-Cog); Clifton Assessment Procedures for the Elderly Behaviour Rating Scale (CAPE-BRS); Clinical Dementia Rating (CDR); Cornell Scale for Depression in Dementia; Holden Communication Scale (HCS); Rating Anxiety in Dementia (RAID)	Intervention group had significantly improved relative to the control group on the following: MMSE (*p* = 0.044) ADAS-Cog (*p* = 0.014) QoL-AD (*p* = 0.028)	The results compare favourably with trials of drugs for dementia CST groups may have worthwhile benefits for many people with dementia

Woods et al. [11], “Improved Quality of Life and Cognitive Stimulation in Dementia”	To examine whether or not improvement in QoL following CST is mediated by change in cognitive function To identify whether there are any specific domains of QoL which change in response to CST	Uses data from study by Spector et al. [10]	Uses data from study by Spector et al. [10] Statistical Analysis on association between baseline quality of life and clinical and demographic variables; predictors of change in quality of life between baseline and follow-up; change in quality of life in relation to change in other clinical variables; change in quality of life and cognitive change-mediation analysis	Improvement in QoL was associated with being female, low quality of life at baseline, reduced depression, and increased cognitive function Changes in cognitive function mediated the effects of treatment in improving QoL	QoL in dementia appears to be independent of level of cognitive function, and interventions aimed at improving cognitive function can, nonetheless, have a direct effect on QoL

Knapp et al. [13], “Cognitive Stimulation Therapy for People with Dementia: Cost Effectiveness Analysis”	To investigate the cost effectiveness of an evidence-based CST program for people with dementia as part of RCTs	Multi-centre RCT with baseline, post-intervention and follow-up assessments *n* = 161 Intervention = 91 Controls = 70	MMSE QoL-AD ADAS-Cog Client Service Receipt Inventory (CSRI) Unit cost Cost of CST	CST has benefits for cognition and QoL in dementia, and costs were not different between the groups.	CST for people with dementia has effectiveness advantages over and may be more cost-effective than treatment as usual

Spector et al. [7], “Cognitive Stimulation Therapy (CST): Effects on Different Areas of Cognitive Function for People with Dementia”	To investigate the effects of CST on specific areas of cognition by an analysis of the subsections of the ADAS-Cog	Uses data from study by Spector et al. (2003).	Uses data from study by Spector et al. (2003) on these measures: MMSE ADAS-Cog	There was a significant difference between treatment and control groups in total ADAS-Cog score (*p* = 0.01) and in the language subscale (*p* = 0.01) There were no significant changes in memory and orientation or praxis	CST appears to have particular effects in promoting language function

Spector et al. [12], “The impact of CST Groups on People with Dementia: Views from Participants, Their Carers, and Group Facilitators”	To investigate whether improvements found in clinical trials were also noted by people with dementia, their carers, and group facilitators in everyday life	Qualitative interviews and focus groups *n* = 38 Dementia = 17 Carer = 14 Facilitator = 7	Framework Analysis.	2 main themes: (i) “Positive experiences of being in the group” (ii) “Changes experienced in everyday life” Overall experience was seen as being emotionally positive and most participants reported some cognitive benefits	Findings supported previous quantitative findings, as well as providing information about the personal experience of CST

Orrell et al. [14], “Individual Cognitive Stimulation Therapy for Dementia (iCST): Study Protocol for a Randomized Controlled Trial”	To develop and evaluate a home-based individual cognitive stimulation therapy (iCST) program for people with dementia which can be delivered by their family carer	Multi-centre, single blind, randomized, two-treatment arm (iCST over 25 weeks versus treatment as usual, or TAU), controlled clinical trial *n* = 306 iCST = 153 TAU = 153	ADAS-Cog QoL-AD Health Survey Short Form-12 (SF-12)	Trial is ongoing	Many people with dementia are unable to access psychological interventions and hence, the development of a home-based individual version of CST will provide an easy to use, widely available therapy package

Aguirre et al. [9], “Cognitive stimulation Therapy (CST) for People with Dementia—Who Benefits Most?”	To investigate which factors may predict response to CST	Pre and Post Intervention assessment *n* = 272 all participants received intervention for 7 weeks	ADAS-Cog QoL-AD Dementia Quality of Life (DEMQOL) Neuropsychiatric Inventory (NPI) Alzheimer's Disease Cooperative Study-Activities of Daily Living Inventory (ADCS-ADL)	Benefits of CST were independent of whether people were taking acetylcholinesterase inhibitor (AChEI) medication Increasing age and female gender were associated with cognitive benefits Care home residents improved more than community residents on quality of life, but the community sample seemed to benefit more in relation to behaviour problems	CST should enhance the benefits for people with dementia who are male and those younger than 80 years

Hall et al. [8], “Cognitive Stimulation Therapy (CST): Neuropsychological Mechanisms of Change”	To examine the effects of CST on specific cognitive domains and explore the neuropsychological processes underpinning any effects	One group pretest-posttest design *n* = 34 all participants received intervention for 7 weeks	MMSE Wechsler Memory Scale-III (i) Information and Orientation (ii) Logical Memory (iii) Visual Reproduction (iv) Digit Span Token Test Boston Naming Test-2 (BNT-2) D-KEFS Verbal Fluency Trail Making	Significant improvement before and after CST group on measures of delayed verbal recall (WMS III logical memory subtest, delayed), visual memory (WMS III visual reproduction subtest, delayed), orientation (WMS III information and orientation subscale), and auditory comprehension (Token Test) No significant changes on measures of naming (Boston Naming Test-2), attention (Trail Making Test A/Digit Span), executive function (DKEFS verbal fluency/Trail Making Test B), praxis (WMS III visual reproduction, immediate), or on a general cognitive screen (MMSE)	Memory, comprehension of syntax, and orientation appear to be the cognitive domains most impacted by CST

Yamanaka et al. [15], “Effects of Cognitive Stimulation Therapy Japanese Version (CST-J) for People with Dementia: A Single-Blind, Controlled Clinical Trial”	To develop the Japanese version of group CST (CST-J) and examine the effects of the program for Japanese people with mild-to-moderate dementia in long-term residential care settings	Single-blind, controlled clinical trials *n* = 56 Intervention = 26 Controls = 30	MMSE Neurobehavioral Cognitive Status Examination (COGNISTAT) QoL-AD EQ-5D Face scale	Significant improvements in COGNISTAT and MMSE for treatment group compared with the control group (*p* < 0.01) EQ-5D was significant (*p* = 0.019) and the QoL-AD showed a positive trend (*p* = 0.06) when rated by care workers. Nonparametrical analysis: improvements in the face scale for mood when rated by both the participants (*p* < 0.01) and the care workers (*p* = 0.017)	The CST-J shows promising improvements in cognition, mood, and aspects of QOL for people with dementia in Japanese care settings A large RCT is now needed

Aguirre et al. [23], “The Effects of a Cognitive Stimulation Therapy (CST) Intervention for People with Dementia on Family Caregivers' Health”	To investigate the effect of CST on family caregivers general health status of people with dementia living in the community attending the CST intervention	Caregiver of people with dementia who attends standard CST intervention plus either maintenance CST or TAU. *n* = 85 Pre and Post Intervention assessment (3 and 6 months follow up)	EQ-5D SF-12	No evidence for a benefit on the family caregiver outcome measures of the intervention before and after CST groups No significant differences between intervention and control groups for any of the variables considered at any time point	CST's benefit for people with dementia that may not carry over to family carers Future studies need to further explore and compare the effects that CST might bring to family caregivers of people with dementia attending CST

Dotchin et al. [16], “Cognitive Stimulation Therapy (CST) as a Sustainable Intervention for Dementia in Low-Resource Settings: A Pilot Study in Nigeria and Tanzania as Part of the Idea (Identification and Interventions for Dementia in Elderly Africans) Project”	To conduct a pilot study of CST in two sites in Sub- Saharan Africa (SSA) in order to assess feasibility and likely utility of this method of intervention	Pilot study (Pre and Post Intervention assessment)	ADAS-COG (adapted) WHO-QOL BREV QOL-AD Hospital anxiety and depression scale (HADS) NPI World Health Organisation Disability Assessment Scale (WHO-DAS) Barthel index	Feedback from both participants and carers was overwhelmingly positive There were no significant differences in cognitive domains noted as measured by the ADAS-COG, although numbers in this pilot were likely to be too small to detect a difference Provisional results indicate small improvements in the primary outcome measure of quality of life	Initial pilot testing indicates that CST is a feasible intervention for dementia and may result in quality of life improvements for patients and carers in both sites

Yates et al. [21], “Service Users' Involvement in the Development of Individual Cognitive Stimulation Therapy (iCST) for Dementia: A Qualitative Study”	To gain insight into perceptions of mental stimulation from the point of view of carers and people with dementia To ensure that the materials are easy to use, clear, and appropriately tailored to the needs of people with dementia and their carers To assess the feasibility of the intervention	Focus groups and Interviews *n* = 52 Dementia = 28 Carer = 24	Focus group in semistructured style guided by a series of predetermined focus points and questions (manual, workbook, activity, and feasibility) Interviews with people with dementia involved completing 2 iCST activities followed by discussion about their perception towards the activities Interviews with carers were to identify any practical issues that might affect the delivery of the program and to gather data about the quality and appropriateness of the activities and manuals	The importance of mental stimulation was emphasized by carers and people with dementia People with dementia saw activities as a way of “keeping up to date” and spending time in a meaningful way Carers reported benefits such as improved quality of life, mood, and memory The concept of iCST was well received, and both carers and people with dementia responded positively to the first drafts of materials Feasibility issues, such as finding time to do sessions, were identified	The feedback from the focus groups and interviews will be used to further develop and refine the iCST program materials in preparation for a field testing phase prior to a large scale randomized controlled trial (RCT)

## References

[1] American Psychiatric Association (2013). *Diagnostic and Statistical Manual of Mental Disorders*.

[2] Alzheimer's Disease International World Alzheimer Report 2010. http://www.alz.org/documents/national/World_Alzheimer_Report_2010.pdf.

[3] Alves J., Magalhães R., Machado A., Gonçalves O. F., Sampaio A., Petrosyan A. (2013). Non-pharmacological cognitive intervention for aging and dementia: current perspectives. *World Journal of Clinical Cases*.

[4] Clare L., Woods R. T. (2004). Cognitive training and cognitive rehabilitation for people with early-stage Alzheimer's disease: a review. *Neuropsychological Rehabilitation*.

[5] Cognitive Stimulation Therapy An Introduction to Cognitive Stimulation Therapy. http://www.cstdementia.com.

[6] Woods B., Aguirre E., Spector A. E., Orrell M. (2012). Cognitive stimulation to improve cognitive functioning in people with dementia. *Cochrane Database of Systematic Reviews*.

[7] Spector A., Orrell M., Woods B. (2010). Cognitive Stimulation Therapy (CST): effects on different areas of cognitive function for people with dementia. *International Journal of Geriatric Psychiatry*.

[8] Hall L., Orrell M., Stott J., Spector A. (2013). Cognitive stimulation therapy (CST): neuropsychological mechanisms of change. *International Psychogeriatrics*.

[9] Aguirre E., Hoare Z., Streater A. (2013). Cognitive stimulation therapy (CST) for people with dementia—who benefits most?. *International Journal of Geriatric Psychiatry*.

[10] Spector A., Thorgrimsen L., Woods B. (2003). Efficacy of an evidence-based cognitive stimulation therapy programme for people with dementia: randomised controlled trial. *British Journal of Psychiatry*.

[11] Woods B. T., Thorgrimsen L., Spector A., Royan L., Orrell M. (2006). Improved quality of life and cognitive stimulation therapy in dementia. *Aging and Mental Health*.

[12] Spector A., Gardner C., Orrell M. (2011). The impact of Cognitive Stimulation Therapy groups on people with dementia: views from participants, their carers and group facilitators. *Aging & Mental Health*.

[13] Knapp M., Thorgrimsen L., Patel A. (2006). Cognitive stimulation therapy for people with dementia: cost-effectiveness analysis. *British Journal of Psychiatry*.

[14] Orrell M., Yates L. A., Burns A. (2012). Individual Cognitive Stimulation Therapy for dementia (iCST): study protocol for a randomized controlled trial. *Trials*.

[15] Yamanaka K., Kawano Y., Noguchi D. (2013). Effects of cognitive stimulation therapy Japanese version (CST-J) for people with dementia: a single-blind, controlled clinical trial. *Aging and Mental Health*.

[16] Dotchin C., Mkenda S., Olakehinde O., Kisoli A., Paddick S., Ogunniyi A. (2014). Cognitive Stimulation Therapy (CST) as a sustainable intervention for dementia in low-resource settings: a pilot study in Nigeria and Tanzania as part of the idea (identification and interventions for dementia in elderly Africans) project. *Alzheimer's & Dementia*.

[17] Moniz-Cook E., Agar S., Gibson G., Win T., Wang M. (1998). A preliminary study of the effects of early intervention with people with dementia and their families in a memory clinic. *Aging and Mental Health*.

[18] Quayhagen M. P., Quayhagen M., Corbeil R. R. (2000). Coping with dementia: evaluation of four nonpharmacologic interventions. *International Psychogeriatrics*.

[19] Quayhagen M. P., Quayhagen M. (2001). Testing of a cognitive stimulation intervention for dementia caregiving dyads. *Neuropsychological Rehabilitation*.

[20] Onder G., Zanetti O., Giacobini E. (2005). Reality orientation therapy combined with cholinesterase inhibitors in Alzheimer's disease: randomised controlled trial. *British Journal of Psychiatry*.

[21] Yates L. A., Orrell M., Spector A., Orgeta V. (2015). Service users' involvement in the development of individual Cognitive Stimulation Therapy (iCST) for dementia: a qualitative study. *BMC Geriatrics*.

[22] Schulz R., Sherwood P. R. (2008). Physical and mental health effects of family caregiving. *American Journal of Nursing*.

[23] Aguirre E., Hoare Z., Spector A., Woods R. T., Orrell M. (2014). The effects of a Cognitive Stimulation Therapy [CST] programme for people with dementia on family caregivers' health. *BMC Geriatrics*.

[24] Donaldson C., Tarrier N., Burns A. (1997). The impact of the symptoms of dementia on caregivers. *British Journal of Psychiatry*.

[25] Olazarán J., Reisberg B., Clare L. (2010). Nonpharmacological therapies in alzheimer's disease: a systematic review of efficacy. *Dementia and Geriatric Cognitive Disorders*.

[26] Prince M., Bryce R., Ferri C. World Alzheimer report: the benefits of early diagnosis and intervention. http://www.alz.co.uk/worldreport2011.

[27] Kurz A. F., Leucht S., Lautenschlager N. T. (2011). The clinical significance of cognition-focused interventions for cognitively impaired older adults: a systematic review of randomized controlled trials. *International Psychogeriatrics*.

[28] Heine S. J., Lehman D. R. (1997). Culture, dissonance, and self-affirmation. *Personality and Social Psychology Bulletin*.

[29] Kitayama S., Markus H. R., Matsumoto H., Norasakkunkit V. (1997). Individual and collective processes in the construction of the self: self-enhancement in the United States and self-criticism in Japan. *Journal of Personality and Social Psychology*.

[30] Spector A., Orrell M., Davies S., Woods B. (2001). Can reality orientation be rehabilitated? Development and piloting of an evidence-based programme of cognition-based therapies for people with dementia. *Neuropsychological Rehabilitation*.

